# The great Galway x rays case 1904 – the first radiological negligence case in Ireland

**DOI:** 10.1007/s11845-025-04235-5

**Published:** 2026-03-03

**Authors:** Roger J. Derham

**Affiliations:** Discipline of Obstetrics & Gynaecology, School of Medicine, University of Galway, Galway, Ireland

**Keywords:** 1904, Expert evidence, Galway, Medical negligence, Queen’s college, Trial, Verdict against plaintiff, X Rays

## Abstract

One hundred and thirty years ago, on the 8th November 1895, Wilheim Konrad Röntgen isolated “X Rays” by accident. After a month spent validating his discovery he published the preliminary findings as a communication in the *Proceedings of the Würtzburg Physics-Medicine Society* in late December 1895. Röntgen deliberately did not patent his discovery and as a consequence X-Ray technology and its potential clinical application was soon recognised and rapidly adopted into worldwide medical practice. By 1899 there was a functioning clinical X-Ray service conducted by and in the Department of Natural Philosophy (Physics) on the ground floor of the Queen’s College, Galway quadrangle. By as early as April 1896, five months after their discovery, skin burns associated with the use of x-rays were being reported and by 1899 an increasing number of legal suits in France and the United States for negligence from the use of x-rays resulting in harm were being reported. In 1904 the parents of a young boy, whose right knee was x-rayed on seven occasions in the Queen’s College in late 1902 and early 1903 for a lost needle segment and who then developed a significant burn injury of his knee, sued the College and the boy’s medical carers for negligence. Based on the daily, anonymous court reporting of the “Great Galway X Rays Case” contained within the *Galway Express (and General Advertiser for the Counties of Galway, Mayo, Roscommon, Limerick and Clare)* newspaper of the 4th to 11th February 1904 and a special summary *Supplement* in the Saturday 13th February 1904 edition, the conduct, characters and outcome, legal and financial, of Ireland’s first radiological negligence case are discussed. Noteworthy where the trial was concerned was the extensive (and expensive) use of expert medical opinion evidence.

## Introduction

**Fig. 1:**
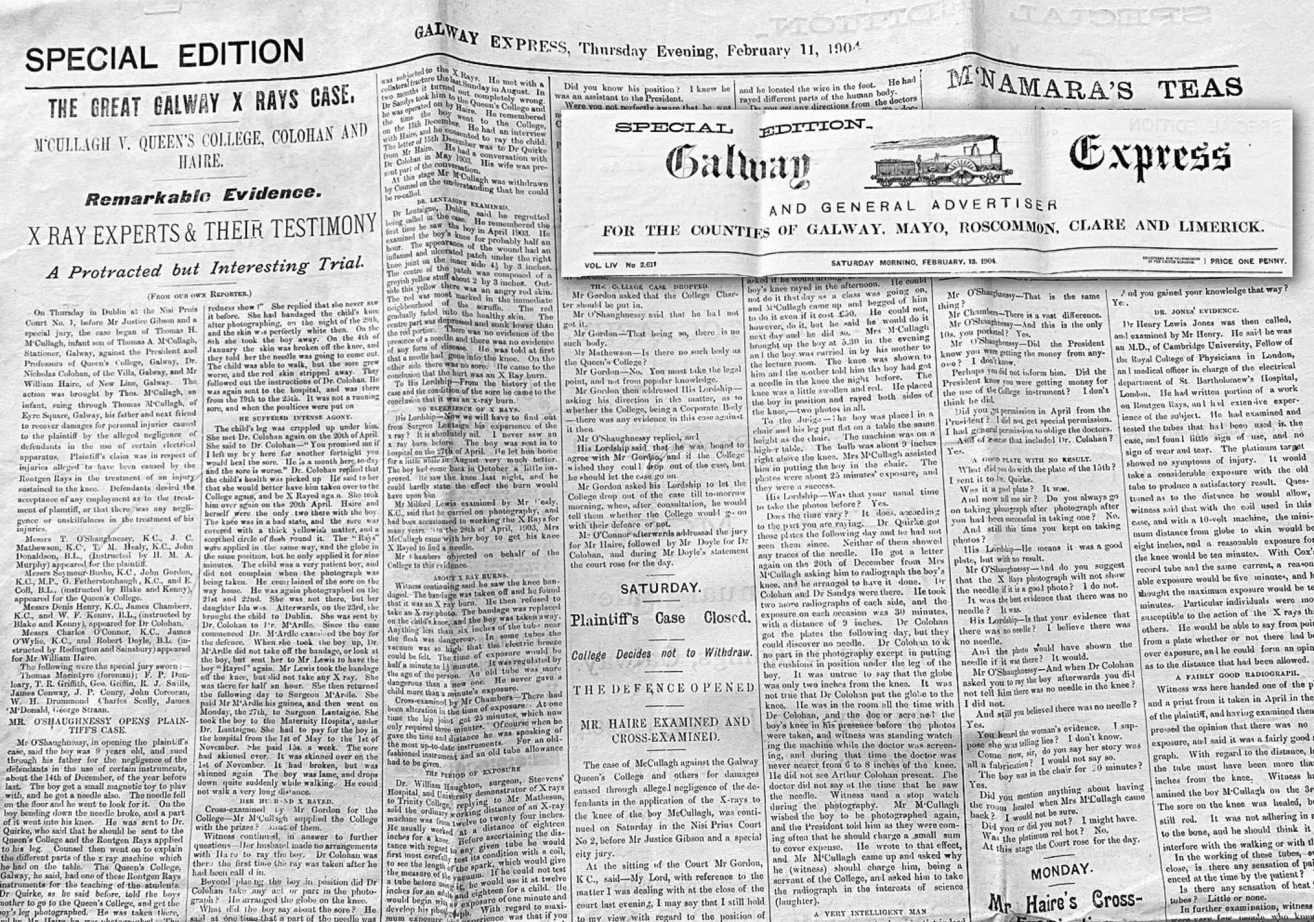
Banner headline of special edition of Galway express newspaper February 11, 1904

This article details and discusses the first medical negligence trial in Ireland in 1904 that involved the clinical use of x-rays. Based on the daily, anonymous court reports of the “Great Galway X Rays Case”, which commenced on Thursday, 4th February 1904 in the Four Courts Dublin. The Court Reports were published in the *Galway Express (and General Advertiser for the Counties of Galway, Mayo, Roscommon, Limerick and Clare)* newspapers of the 4th to 11th February 1904 as well as in a special summary *Supplement* on Saturday 13th February 1904 edition. [1, 2] The conduct, characters and outcome, legal and financial, of Ireland’s first radiological negligence trial are discussed (Fig. 1).

## X-rays and x-ray burns

*“For the sake of brevity I would like to use the term ‘rays’ and to distinguish them from others, the name ‘x-rays’.”*

Wilheim Konrad Röntgen 1896 [3]

On the 8th November 1895, while working alone, as was his custom, Wilheim K. Röntgen, the Professor of Physics in the University of Würtzburg, noticed, while experimenting with electrical charges or “cathode rays”, green florescence occurring on a nearby cardboard panel that was coated with barium platinocyanide. Having worked right through Christmas, much to the annoyance of his wife, elaborating and validating the discovery Röntgen insisted on publishing in late December 1895 a preliminary communication concerning his discovery in a contribution to the *Meeting Reports of the Würtzburg Physics-Medicine Society*. The publication of his communication was unusual in that Röntgen had not first presented a verbal account, as was the custom at that time, of his discovery findings to a meeting of the Society.

By mid-January 1896, news of his discovery was already being announced in mainstream newspapers and as early as the January 23rd 1896 edition of the journal *Nature*, there was a published article by A.C.C. Swinton detailing his own laboratory’s attempts over the previous 3–4 weeks to imitate Röntgen’s work, using information derived entirely from the published newspaper reports [4].

The medical use of the x-ray technology was rapidly adopted into clinical application and yet as early as March 1896 there were already reports of side effects. Thomas Edison [5] stopped working with x-rays in 1900 because of the eye complaints he suffered and the skin cancer that ultimately resulted in the death of his assistant, Clarence Dally in October 1904 [6].

The first published case of an x-ray “burn” appeared in April 1896, and by 1900 at least 170 cases in the U.S. alone had been documented, many being self-reported as being caused by early radiologists [5]. The first Irish newspaper account of the harmful effects of X-rays was a report in the *Dublin Evening Telegraph* reporting a German Medical publication which stated that that ‘Röntgen’s rays burn the skin like the rays of the sun’ [7]. Most of these burns were caused by widespread uncertainty in the duration of exposure and proximity of target in the use of the nascent technology [8] Many early “radiologists” or x-ray innovators died from cancer caused by the radiation burns they self-inflicted in trying to establish the optimal application of the new technology. They are remembered as pioneers in the speciality [9].

By the late 1890’s, 4 years after their discovery, negligence cases alleging injuries or burns caused by x-rays began to be initiated. In Paris, a Mme Mochert sued her radiologist in April 1899 and in the same month in Chicago, Frank V. Belling, a wealthy lumberman, initiated a $25,000 injury suit against a “Roentgen (sic) x-ray laboratory” for burns that resulted in him ultimately requiring a leg amputation [5, 8]. In November 1901 a Dr John Weldon sued the manufacturers of an x-ray device that resulted in him receiving radiation burns. [5] Weldon won the case and was awarded $6750 (equivalent to $254,000 today). Of note multiple experts were utilised in that case.

### An action for medical negligence

To put the “Great Galway X Rays Case” trial in context it is necessary to briefly explain the legal framework in which actions for medical negligence took place in Ireland in 1904. Historically, codified laws such as those contained within the *Hammurabi Code* in Mesopotamia c 1750 BCE, *Solon’s Law Code* in Ancient Greece c. 594 BCE, the Darius I of Persia’s Code of 519 BCE, the Rom *Lex Aquilia* Code of 287 BCE and then the great compendium of Roman Law the *Corpus Iuris Civilis* [10] issued by the Roman Emperor Justinian I in 533 AD included provisions for compensating or punishing negligence including medical negligence [11, 12]. What differed over time was how the codes, particularly the persuasive *Iuris Civilis*, departed from the proscribed corporal punishment of doctors for negligence in Hammurabi, and in general increasingly shied away from penalising doctors unless intentional negligence was evident [13].

There are two major legal traditions in existence and usage. The oldest is the Roman or Civil Law tradition that is operative in most European countries and has its origins in the very earliest codifications of laws such as Hammurabi. The modern understanding of Civil Law began in the early Middle Ages when the Justinian *Iuris Civilis* was “rediscovered” and transmitted to inform and subsequently enable the codification of Germanic law including Salic, Frankish and Lombard common-law procedures and judgements. This process then culminated in the continental European late nineteenth century civil law codes such as the 1900 German *Bürgerliches Gesetzbuch* (BGB) and 1804 Napoleonic *Civil Law codes* [14].

In contrast the Common Law tradition, that evolved and pertains in the main in the United Kingdom and in former colonial and Commonwealth dominions including Ireland, is essentially the consequence of a Norman administrative legal determination, following the 1066 CE Conquest, to manage the *wergild* and blood feud retribution based legal processes in the volatile Danelaw and Anglo-Saxon communities of their new Kingdom, at a judicial and local level [15].

Common Law has over time evolved a category of application of what is referred to as Private Law, a category which includes Tort Law and Contract Law. Torts, in general, address a civil wrong caused by one party to another and Torts of Medical Negligence, in particular, are a later development of the general writs of Torts of Trespass, where there was an allegation of an action causing immediate and foreseeable harm, which then evolved to include the special writs of Trespass-on-the-Case where there were consequential injuries [13].

In Private Law torts, or delicts actions, arise where there is a negative unexpected outcome and where liability is determined after the outcome. Contract actions on the other hand arise where there is a failure of expected outcome. Liability in this case is determined in advance of the action by the terms of the Contract. Medical liability borrows from both and characteristic of both is that the actions are instigated by the parties themselves and can be settled privately.

In 1767 Sir Francis Buller writing in his reference textbook on trials in *nisi prius* courts (jury trials in the main); specifically in the Chapter on injuries arising from “Negligence of Folly”, established the core principle that, *“Every man ought to take reasonable Care that he does not injure his Neighbour”* and if that injury “*be occasioned by Negligence or Folly, the Law gives him an Action to recover Damages for the Injury so sustained*.” [16] David Ibbetson, the Regius Professor of Civil Law in Cambridge, characterised Buller’s analysis as containing two “principal elements”: “Liability arises not simply through an undifferentiated and undefinable *culpa*, or fault but through the neglect of an antecedent duty; and this duty was defined as a duty to take care” [13].

By the 1830 s the standard of objective reasonability where negligence was concerned was stated in 1838 by Tindal CJ in *Lanphier v Phipos* that,

*“Every person who enters into a learned profession undertakes to bring to the exercise of it a reasonable degree of care and skill.” *[17]

and that negligence did not exist if a person (a tradesman) used such care,

“*as any reasonable man, looking at it, would say was sufficient*” [18].

Much of the legal elaborations of notions of duty and reasonableness from the mid nineteenth century onwards that subsequently established the elements of the common law Tort of Negligence in caselaw were supported by the philosopher Immanuel Kant’s writings. Kant’s *Critique of Pure Reason* was published in 1781, the revised second edition in 1787, the *Critique of Practical Reason* in 1788 and the *Metaphysics of Morals* in 1797. [19] Drawing on a Aristotelian concept of “corrective” equality in the application of the law [20] Kant determined that,

*“Duty is the necessity of acting from respect for the law.”*

and that the,

*“moral worth of the character is brought out which is incomparably the highest of all, namely, that he is beneficent, not from inclination, but from duty” *[19]*.*

David Ibbetson echoes Kant in characterising that the duty demanded in tort arose in mid-nineteenth century practice by operation of or “respect for” the law [13].

Borrowing heavily from breaches of contract laws, general negligence guidance and historical Greek and Roman notions of medical liability from the late nineteenth century medical negligence actions became a more frequently utilised and standalone tort that were informed by the key elements of a duty of care, reasonableness and foreseeability [21]. In the development of the concept of reasonability, where medical negligence was concerned, [22] throughout the 1800 s there was an effort to partially ground a legal test of reasonability in the expectations of locality i.e. a country medic would not be as skilful as a city medic, and the care expected for example in Galway would be less than that expected at the centre of medical education and advances in Dublin. Although first promoted as a defence in an English case *Seare v. Prentice* in 1807 [23] “locality” became an accepted defence in American law but did not gain much traction in English or Irish common law [24].

There are very few Irish trials reported preceding the “Great Galway X Rays Case” documenting in caselaw the application of the Tort of Negligence. Lefroy CJ, in the 1862 Irish Case of *Byrne v. Wilson*, stated that negligence could result from both immediate and resulting, chronic or delayed consequences of a breach of a duty of care [25].

In summary, at the time of the “Great Galway X Rays Case” in 1904, the elements of the Tort or action for Medical Negligence had just begun to be established. However as E Gordon points out “*Negligence law for the majority of the nineteenth century was therefore characterised by pockets of liability, governed by recognised categories of duty*” and that “*Courts were wary of imposing too widely the burdensome duty to take care*.” [26] In order to prove medical negligence however by 1904 it was generally accepted that there must firstly have been established that there was a duty of care owed; secondly that there was a breach of that duty; thirdly, there must be evidence of injury or damage and fourthly, there was a causal link between or as a consequence of that breech and the harm caused [24, 27].

## The action

McCullagh v. Queen’s College and Others.

## The plaintiff

The Plaintiff was a **Thomas H. McCullagh**, a child suing through his father Thomas A. McCullagh a stationer in Galway.

## The co-defendents

**The President, Vice-President and Professors of Queen’s College, Galway.** Queen’s College, Galway was founded in 1845 and was one of three constituent colleges, along with the Queen’s Colleges Belfast and Cork, that were incorporated as Queens University of Ireland in 1850 [28]. The Queen’s University of Ireland was dissolved in 1882 and replaced by the expanded Royal University of Ireland which included University College, Dublin; University College, Blackrock; St. Patrick’s College, Carlow; Maynooth College; The Catholic University School of Medicine; Magee Presbyterian College, Derry and Holy Cross College, Clonliffe [29]. For the first time in Ireland the amalgamated Royal University admitted and graduated women on par with men [28].

**Fig. 2:**
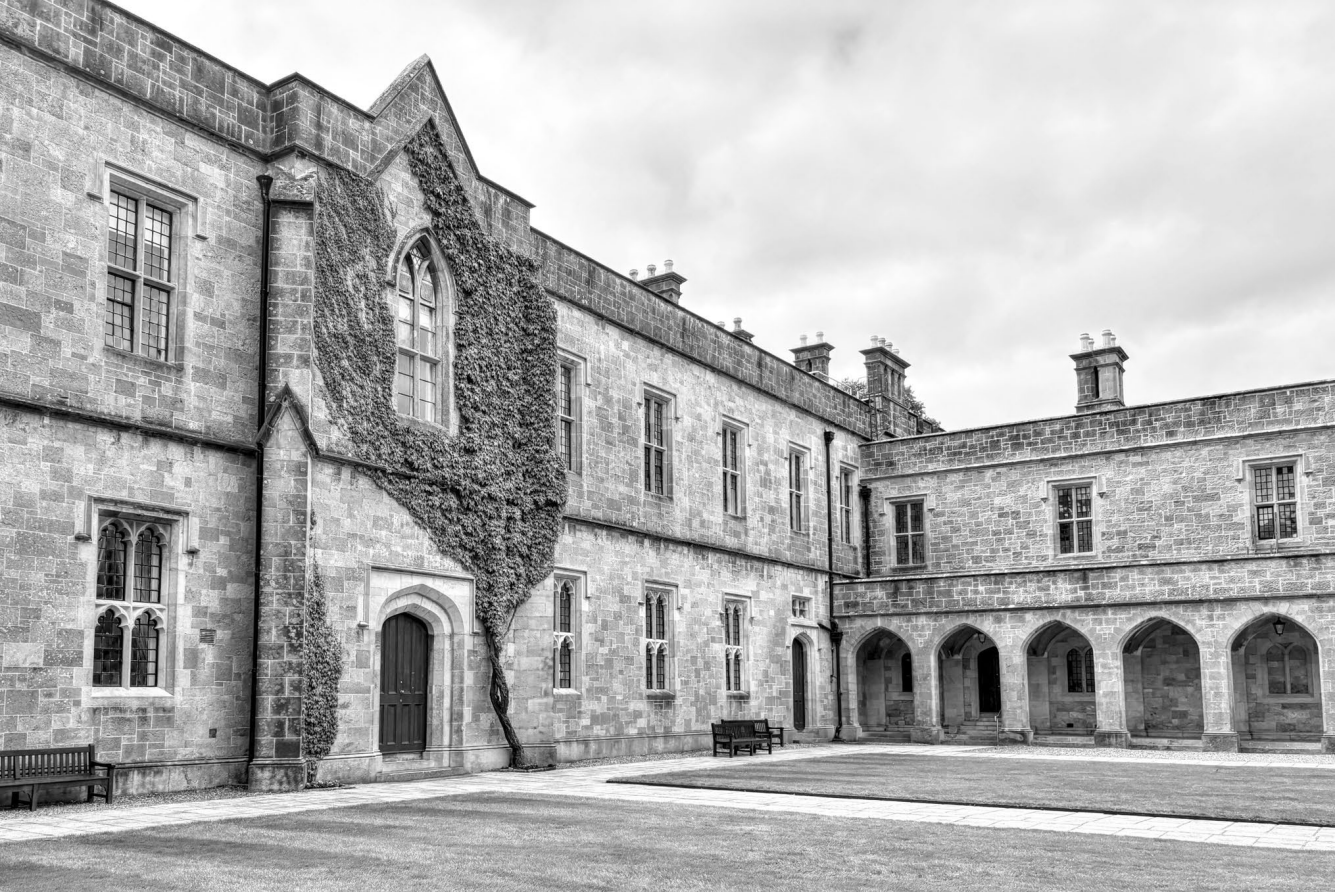
Doorway to department of natural philosophy (Physics), Queen’s College Galway, Quadrangle

The President of Queen’s College Galway in 1904 was Alexander Anderson who was also a graduate of Galway. [30] He was a physicist and the Professor of Natural Philosophy from 1885 −1934. The X-Ray apparatus at the centre of the negligence action was contained and operated within his department. The Physics department was located on the north west corner of the ground floor, abutting the President’s own lodgings, of the College Quadrangle that had been built between 1845 and 1849. Access was through the West doorway of the Quadrangle (Fig. 2).

The reason for naming all the Professors of the College as defendants was uncertainty concerning the governance liability of the College and its Officers under the “new” Charter of the Royal University of 1880. The *Queen’s Colleges (Ireland) Act 1845* established the Queen’s College Galway, along with those in Belfast and Cork. On the 30 December 1845, letters patent under the act were issued incorporating the College under the corporate body name and style of “The President, Vice-President and Professors of Queens College Galway” and in 1850 were re-constituted as the Queen’s University of Ireland by a new royal charter. In February 1882 the Queen’s University was superseded, under the terms of the *University Education (Ireland) Act 18*79, by the Royal University of Ireland. The arguments later made in Court were those related to the nature of the “legal person” of the corporate body of being sued. Was it that defined by the 1845 Queen’s College (President and Professors etc.) Charter or that defined in 1882 Royal University Charter?

**Professor Nicholos Whistler Colahan,** was the son of the Queen’s College foundation Professor in the Practice of Medicine Nicholas Colahan (1806–1890) and joined his father in the medical faculty as Professor of Materia Medica in 1878, remaining in place until 1928, two years before his death. Colahan had been involved in a number of litigations over the years including his election as Surgeon to the County Infirmary with the use of “bogus” governors in 1887 and to a vacancy on the College’s Council in 1896. Although apparently well liked some of his former students felt obliged to disagree. The distinguished surgeon Michael O’Malley said of Colahan, “He was very lazy and spent a good deal of his time on the Corrib. He was quite a neat operator when he took a job in his hands, which was seldom” [31].

**Mr William Haire** had come to Galway in 1894 to teach weaving and design at the Galway Technical School, a school founded at the instigation of a Fr. Lally and funded by the Galway Poor Law Union. The school was initially located in Dominick Street in the town. In 1896 he was appointed a mechanical assistant to the Professor of Natural Philosophy, Professor Anderson by the then President of the Queen’s College Sir Thomas Moffett. From the time of taking up his 1896 appointment he worked on early X-ray technology with Anderson and continued as the main operator of the equipment when Anderson was appointed President of the College.

## The action for negligence

The case involved a suit to recover damages for alleged negligence on the part of the defendants in the “use of certain electrical equipment.” Specifically, the suit alleged injuries caused by “*Röntgen Rays*” to a knee injury sustained by the boy Thomas H McCullagh. The defendants denied negligence. This was the second writ that had been submitted. Originally Dr Colahan had not been included but on advice of counsel he was attached to a revised and resubmitted claim.

## The court, Judge and Special Jury

The *nisi prius* No. 2 Court was an addition in 1837 on the northern Pill Lane aspect of the original Four Courts building, that had been completed in 1802, on Inns Quay, Dublin. The *nisi prius* (unless [tried] before) courts were put on a formal footing in 1285 in England, Ireland and Wales by the Statute of Westminster II allowing for a hybrid of local and central tribunals [32]. By the reforms of 1873 they were a centrally controlled court of the first instance tried before a judge of the King’s Bench Division and, in this particular case, a special jury rather than a “common” jury. The *nisi prius* courts remit evolved through the twentieth century into what we would now encounter in the civil High Court system of today. By a resolution of the judges of the Queen’s Bench (Victoria) Division in 1872, it was declared that there should be three courts of *nisi prius* sitting continuously throughout the legal year, one for special jury cases, one for common jury cases and one, deliberately exclusionary, for non-jury cases. In 1904 at the time of the “Great Galway X Rays Case” trial the High Court of Justice in Ireland included the King’s Bench Division and the Court of Appeal to constitute the Supreme Court of Judicature that had been established by *The Supreme Court of Judicature Act (Ireland) 1877*.

The Judge appointed to hear the case was Justice John George Gibson. Gibson J had been an MP and the Attorney-General for Ireland but resigned both his seat and post when appointed to the High Court in 1888. His elder brother Edward Gibson was created Lord Ashbourne and was Lord Chancellor for Ireland. Gibson J was one of three judges that presided over the “*illegible letters*” trial where Padraic Pearse made his only appearance in court as a barrister [33].

Under the highly legalistic Normans, Saxon law procedures for the use of juries, received formal recognition in Clause 39 of the Magna Carta of 1215 [34]. The independence of the jury from the judiciary as triers-of-fact and the ultimate arbitrators of outcome in jury-based trials was established in common law by the 1670 *Bushell’s Case* [35]. By 1852 in the UK and 1853 in Ireland the use of common juries in civil tort trials was deemed no longer necessary [36] and in the Republic of Ireland formally abolished in 1988 [37].

The “Great Galway X Rays Case” in 1904, in contrast, was heard before a Special Jury. Special Juries evolved from arbitrating on Guild trade disputes in the 1300 s to in later centuries, for example, selecting all female juries who considered disputed cases of paternity or pregnancy. In pursuing or defending a case Special Juries had to be formally requested, and a fee paid and the system was open to very significant abuse [38]. It was also perceived as an educated, elitist system and due to its costs only utilised by or available to the upper or wealthy merchant classes.

The provisions of Sect. 11 of the pre-independence *Juries Act (Ireland)1871* stated special juries would consist of those men (and no aliens or foreigners as allowed in England), “who are the sons of peers, baronets, knights, magistrates, the eldest sons of baronets, of knights, or of magistrates, and of every person appearing in such that as being, either in his own name or as a member of a co-partnership, rated for the relief of the poor in respect of lands, tenements, or hereditaments of the net annual value fixed.” [39] In Ireland’s case this meant the qualifying criteria for a special jury membership from 1872 was a net annual value of property fixed at £50 (€8,486.10 in today’s value) in 1871.

The *Common Law Amendment (Ireland) Act* of 1853 in contrast to the abolishment of common law juries for most civil trials continued to allow for Special Juries to called for by a Judge or by the “Plaintiff in any Action” [40].

The Special Jury selected in the McCullagh “Great Galway X Rays Case” consisted of a Thomas Macintyre (Foreman) along with F.P. Dunleary, T.R. Griffith, G. Griffin, R.J. Swills, J. Conway, J.. Conry, J. Corcoran, W.H. Drummond, C. Skully, J. McDonald & G. Strauss. No specific information about these men is given in the *Galway Express* newspaper court reports.

## Legal representation

The legal teams assembled to contest the Galway case included most of the senior and celebrated lawyers and lawyer-politicians at practice in Ireland at that time including some with a significant scientific or mathematical expertise. Most of the King’s Counsels (KCs) combined legal practice with current or future political positions and most received judicial appointments in the years that followed the case. The cost of this legal expertise did not come cheaply. The fees for a King’s Counsel (of which there were 10) in 1904 would have been about 20 guineas (a guinea = £1 & 1 shilling) brief fee (= € 3600 today adjusted for inflation) and 10 guineas per day refresher. [41] For a junior counsel (of which there were 4) it was 15 and 7 guineas respectively. Solicitors’ fees, of which there were four involved, were about £100 each (€ 18,000 today) for instructing. Total legal costs, not including court fees and expert witness expenses were approximately, for a seven day action, was approximately £1,554 or €282,535 in today’s value. The rules concerning costs were governed by Rules of Supreme Court, Ireland [42].

### Legal representation for plaintiff: T.H. Mccullagh [43].

*Timothy Michael Healy* (Tim) was a nationalist politician who was in the Irish Parliamentary Party under Charles Stuart Parnell and whose mother died in childbirth when he was four. He subsequently fell out with Parnell over Parnell’s attempt to have Captain William O’Shea, the separated husband of Parnell’s mistress Katherine O’Shea, imposed on a by-election seat in Galway. In 1904 he was MP for North Louth constituency representing his own party, the Healyite or Independent Nationalists. He was a controversial and belligerent politician who built up a large legal practice in Nationalist circles. He became the Governor General of the nascent Irish Free State in 1922 and moved into the Vice Regal lodge, now Áras an Uachtaráin. He retired from public life in 1928, published his memoirs the same year and died in 1931, aged 75 [44].

## Legal representation for co-defendant: President, Vice-president and Professors of Queen’s College Galway

Seymore Bushe K.C., J. Gordon K.C. M.P., G. Fetherstonhaugh K.C. & E. Coll BL (Instructed by Blake & Kenny Solicitors, Galway Est. 1892).

*Seymore Coghill Hort Bushe* K.C. (1853 −1922) was a noted orator but often too verbose when defending his clients. Judges were known to interrupt him, to hurry him along. His real life character appears in *Ulysses* on a number of occasions as a result of James Joyce, then aged 17 years of age, having watched him in action successfully defending Samuel Childs in the famous 1895 Childs’ fratricide case [45]. In the *Ulysses* chapter *Circe* for example Joyce observes as a narrator that his character JJ O’Molloy, a “mighthavebeen” lawyer, in his speech “assumes the avine head, foxy moustache and proboscidal eloquence of Seymore Bushe.”

In an analysis of the legal personae and cases in *Ulysses*, the former Supreme Court Judge Adrain Hederman noted that in the Child’s case it was Tim Healy (appearing for plaintiff in “Great Galway X Ray” trial) who did most of the heavy lifting in the defence, an effort that resulted in Childs being acquitted [46]. Seymore Bushe was the grandson of the former Lord Chief Justice of Ireland Charles Kendal Bushe but he himself was later denied a judgeship because he had been sued at an earlier point in his career for “criminal conversation” (the tort of adultery seeking compensation for usurping husbands exclusive rights over his wife) by eloping with a married woman Kathleen Brooke [47].

*John Gordon* K.C., M.P. was the sitting Liberal Unionist MP for South Londonderry during the McCullagh case in 1904 losing out for the same seat to Dr. Colohan’s defence counsel Denis Henry in 1916. Like O’Shaughnessy for the Plaintiff Gordon had also been educated in Queens College Galway. He brought a scientific expertise to the action having graduated in mathematics in 1873 and law in 1876. He was noted for being fair and concise in his examination and arguments. He was appointed Irish Attorney General in 1915 and a judge of the High Court the following year [48].

*Godfrey Fetherstonhaugh* K.C. was appointed Q.C. 1898 and worked mainly on Connaught circuit. He became the Irish Unionist MP for North Fermanagh from 1906–1916 [49].

## Legal representation for co-defendant: Dr Colahan

D. Henry K.C., J. Chambers K.C., & W.F. Kenny BL (Instructed by Blake & Kenny Solicitors, Galway. Est 1892).

*Denis Henry* was a Catholic Unionist, who had a very distinguished undergraduate career and who was made a Q.C. in 1896 at the very early age of 32. He was elected MP as the Unionist candidate for South Londonderry (Derry) in 1916 before resigning his seat to become the first Lord Chief Justice of Northern Ireland in 1921. He was awarded an LL.D by Queen’s College Belfast in July of the same year and he was knighted in 1922 [50].

*James Chambers* had been appointed a King’s Counsel in 1902 and served mostly on the north-eastern circuit. He became a staunch Unionist MP for South Belfast from 1910 to 1917 when he became Solicitor-General for Ireland. He was one of the first signatories of the Ulster Covenant, the protest document signed by about 500,000 people objecting to home rule for Ireland, in 1912 [51].

### For co-defendant: William Haire

C O’Connor K.C., J Owens Wylie K.C. & R Doyle BL (Instructed by Redington & Sainsbury Solicitors, Dawson St., Dublin).

*Charles Andrew O’Connor* K.C. was appointed QC in 1894 and became firstly Solicitor-General for Ireland in 1909 and in 1911 Attorney-General. Based on his judgement in a famous *habeas corpus* case of *R (Egan) v. Macready*, where he ruled that the imposition of martial law by *Restoration of Order Act 1920* did not allow for the death sentence to be imposed [52]. He was only one of two senior judges of the English *ancien régime* to retain their status on the new Supreme Court of the Irish Free State in 1924. [53] He died in 1928.

*James Owen Wylie K.C.*, from a helpful scientific perspective where the physics of the case was concerned, like John Gordon K.C. was a trained mathematician receiving his MA in 1868 in the sister college Queen’s College, Belfast. He was made Q.C. in 1895 and later, in 1914, appointed one of the Lord Justices for Ireland [54].

## Conduct of the Case

The trial opened on Thursday Feb 4th,1904 with an opening statement by Thomas O’Shaughnessy K.C. counsel for the plaintiff. Opening statements by counsel in English jury trials were well established by the 1500 s and their formula detailed by Blackstone in his Commentaries on the Laws of England in 1765. Lawyers in the late nineteenth century, and today, were and are trained to present opening statements as a critical aspect of engaging the jury’s attention to their arguments in a jury trial [55, 56].

O’Shaughnessy K.C. outlined the facts of the case surrounding the burning of a now 9-year-old boy’s leg, in December 1902 in the Queen’s College Galway by “Röntgen Rays”, appraising the jury of the key elements, in a general way, of the plaintiff’s action. O’Shaughnessy had the X-ray equipment on the table in front of him as an exhibit and went about explaining the workings. He then concluded his opening statement by telling the jury that the boy’s leg “was practically burned to the bone” and that he “bore the fruits of that negligence to the present day.”

## Examination-in-chief of Mrs McCullagh, plaintiff’s mother

The First witness for the plaintiff was Mrs McCullagh, the plaintiff’s mother. She was examined by O’Shaughnessy. She stated that her son, Thomas H. McCullagh was 7 years and 9 months old when part of a broken needle from a magnetic toy had apparently lodged in his knee while he played on the floor in December 1902. The following day he complained of pain in his knee and she took him to a Dr Quirke. They both could see a “little” puncture wound on the outside of his right knee. Dr Quirke gave her a letter for the Queen’s College requesting “Röntgen Rays” to be “applied to his leg”. It was her son who brought the letter to the College himself. Mr William Haire, the x-ray technician in the College, demanded a fee of 5 s (€44.85 today), allegedly instructed to do so by Professor Anderson. The fee was agreed-to and the first X-ray was performed on December 22nd, 1902. Mrs McCullagh said that she had held her son for the process and the exposure took 20 min. The image obtained she described as a “blur”. Dr Quirke later when she returned to him said where her son was concerned to “leave things alone.”

Thomas however continued to complain of pain. As Dr Quirke was unwell and unavailable he was then seen at Mrs McCullagh’s request, at home, by Dr Colahan on December 23rd, 1902. Dr Colahan ordered a metal splint and borax lotion to be applied to the knee.

Dr Colahan reviewed the boy again on Christmas Day and St Stephens day (December 26th, 1902) when he said to the McCullagh’s that he would like to repeat the X-ray himself. According to the boy’s mother Dr Colohan “ordered” the boy to attend the College on the following day, December 27th, 1902. Mrs McCullagh in her evidence stated that on that day Dr Colahan had taken the first x-ray and that William Haire had then performed a second screening, the boy’s third x-ray in total. The photographs appeared again to have been of poor quality and later Haire came to McCullagh house and asked that the Thomas come for yet another screening on December 28th, 1902, a fourth. Mrs McCullagh reported that during this screening she saw the globe of the “ray machine” which was positioned close to the boy’s knee get as “red as burning coal”.

On the evening of the December 28th, 1902 Thomas McCullagh (jnr) was no better and his mother arranged with Dr Colahan to have him taken to the hospital the following morning [57]. On admission there he was noted to have redness and what appeared to be a burn on his leg. He remained in hospital until the January 8th, 1903 when Mrs McCullagh said that she “took him out”. At home his leg injury became worse and was weeping. As there was no evidence of the burn healing he was readmitted to the hospital on two more occasions; from the 19th to January 25th,1903 and again for most of March and early April 1903 [58].

On April 20th, 1903 Mrs McCullagh said she had reminded Dr Colahan that he had promised that the boy’s condition would improve whereas it had got worse. Dr Colahan suggested that he be x-rayed again. This was arranged for the College that afternoon and she met Mr Haire there. The boy’s knee had an open circular sore with a red rim and thick yellowish matter on it. The “Rays” were applied for only 9 min, “this time” she said. Thomas was x-rayed again on the 21 st and April 22nd, 1903, amounting to a total of seven exposures since December 1902. Mrs McCullagh said she was unable to come with Thomas for the latter two screenings but that her daughter, Ida, had.

With no obvious resolution of the condition Dr Colahan referred the boy to a Dr McArdle in Dublin for the following day. Mrs McCullagh and her son saw Dr. McArdle on the April 23rd, 1903. He did not actually examine her son’s injury but instead referred him to be “Rayed” again by a Mr Lewis. When Mr Lewis took off the bandage and saw the boy’s knee he refused to take another x-ray. They saw Dr McArdle on April 25th,1903 and he referred Thomas onwards to a Surgeon John Lentaigne. Surgeon Lentaigne arranged to admit the boy to a Dublin maternity hospital where he remained a patient in the hospital from the May 1 st to the November 1 st, 1903 at a cost of 15 s per week (approx. €150/week today) [59]. By November the skin of the boy’s knee had healed over but was friable. It broke down again shortly after discharge and then healed over again.

## Cross-examination of Mrs McCullagh

Mrs McCullagh was first cross-examined by John Gordon KC MP for the College. He established from her that on a personal basis she knew William Haire well; that her husband had had an x-ray in the College by Mr Haire in August or September 1902 and that she still had the letter from Haire demanding the fee for the first x-ray on her son.

Mrs McCullagh was then cross-examined by James Chambers KC for Dr Colahan. He established that her husband had never made any arrangements for the boy to be “rayed”, that it had always been at her instigation. He also elicited the fact that her son was not lame in January 1903. She was pressed on this disclosure by Chambers as to whether he was “running about”? She had replied, “he was never running about”. Chambers also asked her was there some confusion as the boy at one point apparently said that he had pulled out the “lost” needle. Mrs McCullagh reiterated, “the needle never came out”. Chambers then went onto to clarify as to whether Dr Colahan was there for the second and third “rays”. Mrs McCullagh confirmed this and the fact that he had placed the “globe” near her son’s knee and that she had paid him 5 guineas, (adjusted for inflation of 2.6% about €900 today) not for the “rays” but for six home visits to her son [60].

The Judge interjected at this point and reminded counsel that she had also paid 8 shillings a week (approx. €80 today) for the hospital stay in Galway Hospital [61, 62].

Mrs McCullagh was finally cross-examined by Robert Doyle, junior counsel for Mr Haire. She confirmed that she knew Mr Haire well, that she was aware he was the “President’s man” and that he took photographs. Doyle then asked her whether Dr Colahan was “very attentive” and she confirmed that he was “attentive at all times” and remained so even after the negligence action was initiated.

## Examination-in-chief of second witness for plaintiff: Ms Ida McCullagh (sister)

The second witness for the plaintiff was Ida McCullagh, the boy’s sister. She was questioned by J.C. Mathewson K.C. for the plaintiff. She explained that she had attended the College with her brother for an x-ray to be taken on two occasions. She reported that on the first day, the April 21 st, 1903, she saw the sore on her brother’s leg when Dr Colahan took off his bandages. She remembered Dr Colohan saying, “Now, Haire, I would like to take one myself” understanding that an x-ray had been taken before she entered the room. She said Haire then “turned down” a switch, the green light came on in the globe which was then 2 inches from her brother’s leg and the exposure lasted for about 9 min.

The following day April 22nd,1903 she then said she again went to the College with her brother. On this occasion Dr Colahan and a Dr Sandys – another Galway GP and future founder of St. Bride’s Nursing home and Surgical Hospital in Galway – were there. She said that day her brother was in crutches and Dr Colahan took off his bandage and placed him in a chair. Dr Colahan then put the “globe” against the knee and she said that her brother had “cried with pain.” She then stated that she had touched the globe “and it was hot”. Haire had left the room at that point. Three photographs were taken in total that day. The first exposure was thirty minutes, the second eight to ten minutes and the third about twelve to thirteen minutes. Concluding her reply she said her brother’s leg was re-bandaged and that he had cried all the way home and appeared in great pain. That evening he “was roaring”, she had emphasised.

Cross-examined by Denis Henry K.C. for Dr Colohan he asked the plaintiff’s sister to confirm whether Dr Colahan had screened the boy. Ida McCullagh replied that he had done so on the second occasion. Henry then asked her about her description of the wound, whether she had seen it, whether somebody and told her to say the words, and what age she was. She replied she had, that she had seen the wound herself, and that she was 14 years.

The Third Witness-to-fact for the plaintiff was a Michael Barrett, an employee of the plaintiff’s father. Examined by John Donaldson, junior counsel for the plaintiff, Michael Barrett confirmed he was an employee of Mr McCullagh, the boy’s father and that he had brought the boy to the College in April 1903. He was met by Mr Haire and President Anderson’s son. They waited for Dr Colahan for two hours. In that time Haire took a “photograph” and when Dr Colohan arrived he took “another”. Barrett confirmed he had seen the boy’s unbandaged knee and thought it “was bad.”

Cross-examined by Robert Doyle, junior counsel for William Haire, Barrett agreed that the distance from Thomas’ knee to the globe and the exposure duration were those suggested to him by Doyle. When asked how he was certain about the exact duration of the exposure, Barrett replied, “I guessed it.” There is no way of knowing but this statement could have been critical in influencing the later deliberations of the jury. In eliciting the response from Barrett, Doyle the junior counsel had managed not only to get Barrett to undermine both his own factual evidence but by extension all factual evidence for the plaintiff by stressing for the jury the possible imaginary nature of it!

The Fourth Witness-to-fact for the Plaintiff was Thomas A. McCullagh the child’s father. Mr McCullagh had a very brief examination before being withdrawn. He confirmed the letter from Haire and that he had consented to the arrangements. He began to explain his own issues with a poorly healed bone fracture and that he had been x-rayed by Haire for a “collateral” fracture in August 1902. At this point O’Shaughnessy withdrew him from the witness box, informing that he could be recalled to the give evidence again later.

The Fifth and Final Witness-to-fact for the Plaintiff – Surgeon Lentaigne. Examined by O’Shaughnessy Surgeon Lentaigne immediately stated he “regretted” being called in the case. He recounted his examination of the boy in April 1903 and his findings of an “inflamed and ulcerated patch” behind right knee on medial or inner side. The centre was depressed and there was no evidence of a needle. He had come to the conclusion, following his examination, that “the hurt” he saw was an “X Ray burn”. The Judge asked O’Shaughnessy to clarify what had the surgeon said. O’Shaughnessy replied that Lentaigne had said that, ‘From the history of the case and the condition of the sore he came to the conclusion that it was an x-ray burn.’ At this point the judge Gibson J asked Lentaigne directly of his experience of x-ray burns. Lentaigne replied it was “absolutely nil!” Again there is no way of knowing for sure but in the jury’s deliberations, the probative value of Lentaigne’s evidence for the plaintiff would almost certainly have been undermined by his admission, although truthful, of his ignorance of x-ray burns, thereby creating doubt as to the actual causation of the injury.

## Expert witnesses for plaintiff

First allowed for the in the 6th Century Byzantine Justinian Code, [63] by the early eighteenth century the notion of expert witnesses giving opinion evidence in jury trials was well settled and accepted. In 1782 the admissibility of expert evidence was formally established in common law in the *Folkes v. Chadd* case – adjudicating on the impact of harbour silting by tidal embankments – and furthermore the case also determined that experts could appear for either party to a case [64, 65].

The first expert witness for the Plaintiff was a Mr Milford Lewis. Mr Lewis’ professional associations or place of work were not detailed in the *Galway Express* newspaper court reporting but he confirmed that he had been taking X-rays for a number of years. When the boy was referred from Surgeon Lentaigne he came with his mother. However when he unbandaged and saw the boy’s knee he refused to do an x-ray. Lewis stated that in his opinion the vacuum tube should never be less than 6 inches from the skin, that an exposure time of 1–1_1/2_ min was enough and dictated by age of patient. He also maintained that “an old tube was more dangerous than a new one”.

Milford Lewis was then cross-examined by James Chambers K.C. for Dr Colahan. Lewis clarified that the exposure times he detailed were those that are current and since the introduction of x-rays exposure times had changed particularly with new and improved equipment. Exposure times were very different with the older equipment and “allowance had to be given.”

The second Expert Witness for the Plaintiff was a Dr William Steele Haughton. Haughton was a Surgeon to Steevens’ Hospital, Dublin and a University Demonstrator in Röntgen Photography in Trinity College Dublin. [66] He was an early user and advocate for the “new” X-ray technology in medical practice having purchased his first x-ray unit in London in March 1896. He had presented an early paper on his work with X-Rays in March 1897 [67].

Haughton was asked to explain the ordinary working distances of an x-ray machine and replied that he would set it at 12 inches for an adult and 18 inches for a child and generally he found that a maximum exposure for about 5 min was enough to obtain an adequate photograph.

At this point the court “rose” (adjourned) for the day and re-convened the following morning, Friday 5th February 1904 when D Henry K.C. for Dr Colahan cross-examined Dr Haughton.

Dr Haughton was first asked to explain for the jury the difference in burns caused by low-voltage or high-voltage tubes. He was also asked whether the tube used on the plaintiff, from his examination of it, had been damaged or interfered with. Dr Haughton replied that it would be impossible based on his recent “sight and touch” of the tube to be certain “without testing”. It was notable that Gibson J stepped in at this point and asked for specific clarity from the witness as to whether on “sight and touch” there was any evidence of “improper use”, as distinct from damage or interference. Houghton replied “No.” Gibson J then proceeded, rather than counsel, to interrogate Dr Haughton in a series of specific questions, to detail the pathophysiology of the causation and timeline of burns formation. The judge established the distances that he would normally use. Importantly he also asked whether Dr Haughton had ever caused a burn with his clinical application of x-rays? Houghton replied “No!”.

Following this judicial interjection Henry K.C. read out a passage from a scientific article which discussed lesser degrees of burning, from x-rays. The Judge asked whether the witness agreed with the read passage. The witness replied “Yes”. Henry K.C. said “no wonder” as the passage had been written by Haughton himself. He finished the cross examination asking whether some people were more susceptible to X-ray burns than others. Houghton replied, “The susceptibility is not yet proven. It is very difficult the application of X-rays”, a spontaneous observation from an expert witness for the plaintiff that would embolden the defence to later emphasise for the jury the notion of “ordinary skill” being applied in the x-rays taken of the plaintiff.

The Third Expert Witness for the Plaintiff was a Dr Richard Lane Joynt. A cousin of Lady Augusta Gregory of Coole Park in Galway, Lane Joynt was a trained orthopaedic specialist who became one of the first Radiologists appointed in Ireland when he took up a position in the Meath Hospital in Dublin in 1900. As a result of his experimentation and work with X-rays he had burnt his own hands to the extent that they were always bandaged. In giving evidence Lane Joynt never referred to the burns of his own hands caused by X-rays nor was he asked about them, something the plaintiff’s or defence counsel either deliberately or by omission avoided pursuing. On the other hand it was, perhaps, an intentional decision of the plaintiff’s solicitors, to seek Lane Joynt’s expert opinion as he was also, at the time of the trial, the medical officer of the Society for the Prevention of Cruelty to Children [68].

In response to questioning by counsel he said he had recently examined the boy Thomas McCullagh (jnr), just before the trial began. Lane Joynt said he agreed entirely with Dr Haughton’s evidence in regard to the distances of vacuum globe and length of exposure and that he also had concluded it was an x-ray burn.

Cross-examined by Chambers K.C. for Dr Colahan, Lane Joynt he said it was the first “ulcer” he had ever seen.

At this point Thomas McCullagh (snr), the boy’s father, was recalled to the witness box and cross-examined by Henry K.C. on the authorship of a letter he presented McCullagh with. McCullagh said it was in his wife’s handwriting. Henry demanded to know whether the letter contents were with his authority knowing full well that Mrs McCullagh was very much involved in requesting and overseeing the care for her son. Thomas McCullagh replied that his wife “took the matter into her own hands completely.” Henry K.C. pressed him on the point and asked whether it was with his authority. He replied “Yes” [69].

Henry K.C. then asked Thomas McCullagh (snr) about another letter from the family solicitor Mr Henry Murphy to Dr Colahan notifying him of the intention to sue and apologising on his and Mr McCullagh’s behalf by expressing regret at having to do so. Thomas McCullagh was then cross-examined by Charles O’Connor K.C. for Mr William Haire. He was asked whether had he known Haire for a long time and had he known of Haire’s “humble” position in the College. McCullagh described Haire as an “assistant to the President.” The Judge interjected and asked him did he not know that Haire was a just a “superior porter” in the College? McCullagh denied knowing this but stated that he knew Haire as a “very intelligent man” who had come to Galway originally as an “instructor in weaving”. [70] The judge seemed incredulous and asked, “You knew that he came to Galway as an instructor in weaving?” McCullagh replied “not at that time” (Dec 1902) but only in the past 2 months, and that “a Fr Lally had told him”. Fr Lally was the founder of the Technical School in Galway where Haire was first employed as a weaving instructor.

## Legal arguments in regard to legal status of College

Thomas McCullagh senior was then due to be cross-examined by Gordon K.C., M.P. for the Queen’s College but before questioning of the witness could begin a great deal of legal argument ensued over the actual legal status of the Queen’s College and whether it was appropriate for the College to be sued. Gordon asked that the Charter of the College be entered into the record. O’Shaughnessy K.C. immediately stood up and said he had not got sight of it. Gordon then asked the judge to dismiss the case against the College, being a Corporate Body, unless there was “any evidence” against it. O’Shaughnessy pouted and said “Any?” sarcastically. Gibson J said he agreed with Mr Gordon and that the College could drop out of the case if it wished and that the remainder of the case would proceed. Gordon said he would consult with his clients and let the Judge know the College’s decision the following morning.

## The Defence case

The plaintiff’s case at that point was declared concluded and thus it was time for the defendants to begin theirs. Before the court rose for the day O’Connor K.C. made an opening statement for the first defendant Mr Haire and Henry K.C. began but did not complete an opening statement for Dr Colohan.

The morning session on Saturday February 6th,1904 opened with Gordon K.C., M.P. informing Gibson J that the Queen’s College would not withdraw from the case, because there was a danger the trial would then be put aside or abandoned for the other defendants, which they did not wish to happen.

Henry K.C. then recommenced his opening statement on behalf of Dr Colahan. In addressing the special jury he called the action against his client “almost an afterthought” and said it was reasonable to refer the boy, Thomas McCullagh (jnr) to Mr Haire who had expertise in x-ray photography, rather than incurring expense for the family by referring the boy at the outset to Dublin. He also said that the boy contributed to his own poor outcome by running about despite “instructions” during his convalescence to the contrary.

Henry K.C. then informed the court about his intention to call a number of distinguished expert witnesses in X-Rays who between them had not seen more than 4 or 5 burns and reminded the jury that Dr Colahan’s advice (in regard to the boy having the “rays”) was according to his best judgement and was that of a “country practitioner in respect to a new science which was only in the experimental phase”. The standard the jury would have to examine, he stressed, was, using a locality argument, was “what was the average degree of medical knowledge and skill that they should expect in the case?”.

## Examination-in chief of co-defendants

### Mr William Haire: X ray technician

Mr William Haire was the first co-defendant called to give evidence. He was first questioned by his own counsel James O. Wylie K.C. and said that he was employed in the Queen’s College, Galway as a “mechanical assistant” to the Professor of Natural Philosophy, President Anderson. The judge, Gibson J then interjected and asked him what his salary was? He informed the court that his salary was £58 per annum (equivalent to €7212 today). Gibson J then asked Haire whether he had other “emoluments” or accommodation support, to which Haire answered “No”. He stated he had been in position since 1896, and for the previous 8 years (1896–1904) they had been using “the X-rays” in the Department of Natural Philosophy (Physics).

Haire then gave evidence that the equipment on display in the court was the equipment they had used in the College up to April 1903. He had seen Professor Anderson, his former teaching assistant Mr Henry, and a Professor Townsend from Oxford as well as some of the students use the equipment on many occasions. He said that before x-raying the plaintiff for the first time he had used the equipment previously to take x-rays on about 50 occasions. He normally did not accept a gratuity for doing so but some of the “local doctors” had paid him on occasion. He had been paid once previously by a Dr Sandys and once by the Governor of Galway Prison before Easter of 1903. Haire then confirmed that he began the x-ray “work” when the previous demonstrator Mr Henry had left. Gibson J asked whether he was doing it for “profit” or to “oblige the doctors. Haire replied “just to oblige the doctors.” He said his first patient was a private one, “to locate a piece of wire in a foot.” The judge asked was it a “success”? Haire said that he had located it.

Turning to the case being tried Haire recounted getting a letter, which he had not kept, requesting an x-ray for the plaintiff. He could not do it that day. The plaintiff’s father had come up to the College and begged him to do it, “even if it cost £50”. He said he could not as there was “a class” going on but would do it the following day. “He did so,” Haire then emphasised, referring to Thomas McCullagh coming to the College. He said that Mrs Mc Cullagh had come with the boy. It was she who had carried her son into the lecture hall where the equipment was set up. Haire said then that he had positioned the boy and took “two photos in all”. He said the knee was a “little swollen and red” when he looked at it before starting. In describing the procedure process in more detail, after being prompted by Wylie, Haire said that the boy’s mother had held him for the exposure, that the bulb was about 9 inches “above” the knee and that the exposure lasted about 25 min. He said, “they were a success.” Gibson J then asked whether that was his “usual time”?. Haire answered that “it was”. When asked what happened to the photographic plates from that first day, he replied that Dr Quirke had got them and he had not seen them since. He then said that in his opinion there was no evidence of “the needle” in the photographs.

Haire then went on to recount that he got a second letter concerning the boy, this time from Mrs McCullagh, and arranged to x-ray him again on December 27th, 1902. Dr Colahan and Dr Sandys were present for this exposure. He said that he had taken two more x-rays on this occasion, one on either side of the knee, that the bulb was never closer than 9 inches and that the exposure for each was 30 min. He also emphasised that Dr Colahan had taken no part in photography apart from positioning the child. When the photographs were developed again there was no evidence of a needle. Later Mr McCullagh contacted him again asking him to take yet another “ray”. It was at this point, given the number of x-rays that had been performed, that Professor Anderson suggested a small fee for the service. Mr McCullagh balked at the request and had asked Haire whether he might just take the radiograph “in the name of science.”

O’Shaughnessy K.C., lead counsel for the plaintiff Thomas Mc Cullagh (jnr), interrupted by saying sarcastically, “A nice scientist indeed!”. The judge Gibson J, obviously irritated by O’Shaughnessy’s interruption, then said pointedly for the special jury’s benefit, “I think witness is a very intelligent man.”

Haire went on to deny that the vacuum tube used in the April 1903 x-ray was red hot, as alleged by the plaintiff’s sister, as he had installed a new more efficient tube prior to that x-ray. Because of the new tube the exposure was reduced to 5 min and conducted at a distance of 12 inches. Gibson J again interjected and asked Haire whether the x-ray work was hard? Haire replied “No, my Lord.” To laughter in the Court the judge then asked, “I suppose you are paid for the quality, not the quantity?” Haire said “Yes” and in response to a further question from the judge and more laughter in the court said he was not paid “enough” for the work that he did.

## Cross-examination of first defendant Mr William Haire

William Haire was then cross-examined by T O’Shaughnessy K.C. for the plaintiff in what the newspaper report described as a “lively” cross-examination. It began by an interrogation of Haire as to who had given permission for the X-ray to be taken, who had requested the fee for same, and whether the College had had given permission. At that point Seymore Bushe K.C. stood up and objected to any actions of the College as a corporate body being examined in Haire’s evidence. O’Shaughnessy, ever prickly, said that if he, “was not allowed to question the witness he might as well sit down.”

Gibson J stepped in at this point and said he would allow the line of questioning but any evidence could not be entered against the College because he, Gibson J, did not think the “President” as a legal entity had any right to give permission. Here Gibson J appeared more determined than ever to remove the Queen’s College from any possibility of liability even if his offer for them to be removed as defendants in the case was turned town. He deliberately distinguished for the special jury Professor Anderson’s role as Professor of Natural Philosophy in whose department the X-ray equipment was held from Professor Anderson’s role as President of the Corporate Body known as Queen’s College Galway.

Haire was then questioned about how he got the money for the X-rays and this line of enquiry caused O’Shaughnessy K.C. to clash with J Chambers K.C. for Dr Colahan over the difference between getting money directly or encashing a cheque that had been made out to and given to Professor Anderson. O’Shaughnessy then asked Haire directly whether Professor Anderson knew that he often got money for using College equipment. Haire answered, “I don’t think he did”.

Following directly on from the monetary angle O’Shaughnessy K.C. then moved to questions about the X-rays themselves. He asked Haire why he continued to take X rays of Thomas McCullagh (jnr) when he had given evidence that the original “plate”, taken at the first instance on December 15th,1902, was “a good one?” Gibson J again interjected to address O’Shaughnessy directly, “He (Haire) means it was a good plate but with no result.” O’Shaughnessy persisted and asked Haire was it his evidence that based on the first photographs that there was no evidence of a needle in the knee? Haire said “Yes” but also stated that the photographs were not conclusive proof. Gibson J asked Haire to again clarify, “Is that your evidence that there was no needle?” Haire replied, “I believe there was no needle,” a reply which was not challenged by O’Shaughnessy allowing the special jury to accept Haire’s evidence as that of an expert witness.

O’Shaughnessy K.C. then asked Haire whether he had informed Dr Colahan that he thought there was no needle. Haire said he “did not.” O’Shaughnessy then asked did he believe Mrs McCullagh’s evidence. “I suppose she was telling lies?”, the counsel suggested sarcastically. Haire replied, “I don’t know.” O’Shaughnessy persisted, “her story was a fabrication?”. Haire replied “I would not say so.” At that point the judge called a halt to proceedings for the day and the Court rose for the weekend.

One significant responsibility of O’Shaughnessy was to undermine Haire in the eyes of the special jury, either by examining him on the evidence presented such as the facts or technical matters for obvious fault-lines, or by undermining him as an evidence giver i.e. experience, honesty, understanding of duty etc. O’Shaughnessy interrogated along both lines of attack. The initial part of the cross examination was designed to imply Haire was defrauding the College by demanding fees for the X ray work they were unaware of, and therefore not to be trusted, least of all by the jury.

The *Galway Express* reported that the trial reconvened on Monday, February 8th,1904 and that William Haire’s cross-examination by T. O’Shaughnessy K.C. for the plaintiff Thomas McCullagh (jnr) re-commenced. Haire was asked to recall the events of April 20th, 21 st & 22nd 1903 and to state exactly how many photographs were taken. Haire answered in relation to the 21 st that he only took 1 photo that day. O’Shaughnessy addressed the jury as he spoke, “Yes”, he dismissed before turning to Haire. “But you do know one plus one makes two, even in Galway.” O’Shaughnessy then went through a serious of technical questions which Haire answered competently. O’Shaughnessy then finished this part of his examination by asking Haire, “Did you know there was a danger of burning in X-ray work?” O’Shaughnessy did not specify whether the burns he was referring to were to oneself or to a patient having x-rays. Haire answered emphatically, “Yes”.

It is interesting that O’Shaughnessy did not, according to the court reporting, not examine Haire’s admission that he was aware of burns being caused by X-rays further. Barristers would shy away from a technical examination of key defence witness or defendant if they were not expert enough, lest a witness’ accomplished technical evidence under cross-examination hinder their intent to undermine the same witness in the special jury’s deliberations. An additional aspect was that Gibson J seemed determined to protect Haire from O’Shaughnessy’s probing and O’Shaughnessy probably realised this line of attack would be counter-productive if he clashed with the judge. He also knew that he would be able to examine the “burn” issue in questioning expert witnesses for the defence.

O’Shaughnessy then concentrated on Haire’s use of Queen’s College notepaper to present the account for the x-rays, a use for which he would have needed the Registrar’s permission. Haire said it was the President’s personal notepaper that he used. O’Shaughnessy asked, “Did he have permission to use it?” Haire replied “No”. O’Shaughnessy asked Haire was it wrong of him to use the paper. Haire replied “Yes.” O’Shaughnessy drew himself up to his full height, looked at the jury and then at Haire. He asked him was that action of using the President’s notepaper without permission “a violation of duty?” Stung by the accusation, Haire replied, “I would not go that length.”

To finish the cross-examination O’Shaughnessy wanted to locate Haire for the jury in his proper place in the overall scheme of things. He asked about Haire taking up a position in Galway. Haire informed him that he had come to Galway as “a teacher of weaving and designing at the Technical School.” “Not by electricity?” O’Shaughnessy probed. “No”, agreed Haire. O’Shaughnessy then asked about Haire’s appointment as an assistant to the “Professor of Natural History” (sic: should have been “Natural Philosophy”). “Was it to teach weaving?”, he asked Haire with feigned innocence. “No”, replied Haire. “Was it to help him do nothing?”, O’Shaughnessy asked. “No”, Haire replied. “To look after the machines, cleaning?”, O’Shaughnessy continued probing. “Yes”, replied Haire. O’Shaughnessy paused for effect. “And you gained your knowledge that way,” he half-mocked. William Haire replied “Yes!” and his cross examination was over.

## Expert witnesses for the Defence

The First Expert Witness for the Co-Defendants was then called, a Dr Henry Lewis Jones of St Bartholomew’s Hospital in London [71]. Questioned by D Henry K.C., for Dr Colahan, Dr Lewis Jones detailed his experience; he was currently the medical officer in charge of the Electrical Department in St Bartholomew’s Hospital, London and had “extensive experience” of Röntgen Rays. He had been asked to examine the equipment used in the Galway case. He declared it to have little sign of wear and tear or use. Humanising the inanimate nature of the machinery he stated that the platinum target showed “no symptoms of injury”. He then said that because the vacuum tube was old it would require a considerable exposure to obtain a “result.” He then said that with a 10 V machine the globe to skin distance should be a minimum of 8 inches and a reasonable exposure for the knee about 10 min. He volunteered to opinion an estimate of exposure and distance if shown a plate.

The witness was handed one of the plates and a photograph taken from it from the April 1903 exposures. He declared it to be a good exposure and that the globe must have been more than 2 inches from the knee. Dr Lewis Jones then affirmed that he had examined the boy on February 3rd, 1904, just before the trial opened. He said the skin on the knee had healed, although still a little red. It was not adherent to bone and he thought it should not interfere with walking. Lewis Jones was then asked about sensations of pain or heat associated with the use of the X-ray tubes. He answered that there is no association with pain and little or no sensation of heat. Finally, he declared that at that time there were very few people who knew “anything about the working” of X- Rays.

Dr Lewis Jones was then cross-examined by J.C. Mathewson K.C. for the plaintiff. In response to a specific line of questioning Lewis Jones gave opinion evidence that if a patient felt pain during the screening then there “was something wrong with the patient or the equipment” and that to a skilled person, evidence of inflammation would generally indicate overexposure. He was then asked directly by Mathewson would a skilled person have exposed for 25 min one day and a similar exposure time the following day. Lewis Jones replied in the affirmative, “Yes. I have heard of such exposures.” Mathewson persisted, “Would it be dangerous?”. Lewis Jones replied, “It would be risky.”

The Second Expert Witness for the Co-Defendants was a Dr Hall-Edwards. His professional experience was first explored by Chambers K.C. for Dr Colahan [72]. Major Dr John Hall-Edwards was yet another very high-profile expert witness for the defence. He detailed his expertise. A keen photographer he became an early ‘radiologist’. He was the first clinician, on the January 11TH, 1896, to use x-rays in a simulated clinical case (ironically a “lost” sterilised needle buried in his assistant) and a short time later in Feb 1896 was the first to use x-rays to direct a surgical operation. In 1899 he was appointed Surgeon-Radiographer to the General Hospital in Bermingham in charge of the “X-ray and light department”. He had also enlisted and served as the first military radiographer to the Imperial Yeomanry Hospital service during the Boer War in South Africa. Finally, Hall-Edwards disclosed that he was the current editor of the earliest radiology journal the *Archives of the Roentgen Ray* which had previously been called the *Archives of Clinical Skiagraphy* and which had established in 1896. (The *Archives of the Roentgen Ray* ultimately became the *British Journal of Radiology.*) Hall-Edwards also stated that he had devoted the previous 8 years of his professional life entirely to X-rays*.*

Chambers then examined in detail the expert witness’ knowledge of the application and hazards of X-rays. He was asked to give an opinion estimate of ideal distance from the vacuum tube a subject should be and was then asked to proffer his opinion of a photographic plate of the plaintiff’s knee from April 1903. Hall-Edwards was then asked about the exposure times he had utilised in his practice and said they had been long in the “early days”. He was then asked specifically about ever producing a burn and he replied emphatically “Never” [73]. Finally, from a factual perspective to ground the jury in the current reality, he was asked his opinion of the present clinical status of the plaintiff Thomas McCullagh (jnr), the boy whom he had the opportunity to examine before the trial commenced. He said the boy’s skin was tender but it was his opinion that it would harden and “become all right in time”. [74]

The examination of the Third Expert Witness for the Co-Defendants, Sir Thornley Stoker then commenced. Sir Thornley Stoker was called by J Chambers K.C. for Dr Colahan. Sir Thornley Stoker, a brother of the Dracula novelist Bram Stoker, was ironically or perhaps inspirationally, the Inspector of Vivisection for Ireland. A surgeon in Dublin’s Richmond Hospital he was President of Royal College of Surgeons in 1894–1896. At the time of the “Great Galway X Rays Case” he was President of the Royal Academy of Medicine. He was a graduate of Queen’s College, Galway, graduating with an MD in 1866. In 1875 another brother Dr George Stoker presented the results of his experiments conducted, with Thornley Stoker’s encouragement and facilitation in the Richmond Hospital, with oxygen gas therapy for the healing of wounds and ulcers. Of gynaecological interest he performed the first abdominal hysterectomy in Ireland in 1878. He was knighted in 1895. One slight personal quirk was his habit for a time of buying a piece of antique furniture after a successful major operation. Oliver St John Gogarty, a friend of Stoker said of him, “The Aubusson carpet in the drawing-room represents a hernia, the Ming Cloisonné a floating kidney, the Buhl cabinet his opinion of an enlarged liver, the Renaissance bronze on the landing, a set of gall-stones”.

Stoker gave evidence that he employed X-rays in his work and that he also would have done so for a presumed needle lodged in the knee. He then opinioned that the practical workings of X-rays were “not within the ordinary knowledge of an ordinary surgeon.” He also had examined the plaintiff prior to the case commencing and found no connection between the sore on the inside of the right knee and the joint. He was not cross-examined.

The Fourth Expert Witness for the Co-Defendants was a Dr John O’Donnell, Assistant Physician in in the Mater Misericordiae Hospital in Dublin, who was called to the witness stand, by D Henry S.C. for Dr Colohan. He detailed his extensive experience as the head of the Electrical Department (x-rays) in the Mater Hospital and explained the difference in electric current between the older and newer vacuum tubes. He said that he had x-rayed some very young children that had to be sedated with chloroform. He detailed his experience of long exposure times with older tubes of 25–30 min but said that newer tubes were “one-fourth or one-fifth” of that. His globe-skin distance was a minimum of 6 inches and he also had never seen an x-ray burn. [75]

There was no cross-examination conducted on O’Donnell whose evidence and opinion, like Stoker’s were not considered detrimental to the plaintiff’s case. The Court rose for the Day.

## Special jury’s revolt

The fifth day of the “Great Galway X Rays” trial on Tuesday February 9th, 1904 opened with a demand, once the Court had been called to order, by the foreman of the Special Jury, Thomas Macintyre, for an increase in the Special Jury remuneration which had been set at 1 guinea for the trial. In instantly dismissing the application Gibson J said, to laughter in the court, that he had no jurisdiction to increase their fee and the only (extra) benefit from serving on the Jury they could “derive” would be from the “knowledge they were gaining”.

## Examination of second defendant: Dr N Colohan

The examination-in chief of co-defendant Dr N Colahan then took place. Colahan was initially led through his sworn evidence by James Chambers K.C. and said he had first come into contact with Mr McCullagh (snr in relation to the injury to his son Thomas (jnr) on Dec 24th, 1902. He had then attended the “case” subsequently in the College for the taking of the X rays and had positioned the boy with cushions. He gave evidence that the globe was about 8 inches from the knee and the exposure time was about 25 min. The boy had said the needle was in his knee as he could see its thread. Dr Colahan stated that he had examined the knee and determined that the thread the boy was seeing had come from his sock. He then said, “In April (1903) a ray was taken and the needle was shown.” They then did another X-ray to “verify the position for operation” and this time there was no sign of the needle. He emphasised that the “globe” had never touched the boy’s skin and he had not complained of pain. Dr Colahan said he had never received any complaints from the McCullaghs until he got the solicitor’s letter informing him of the action being taken against him.

Dr Colahan was then cross-examined by O’Shaughnessy K.C., for the plaintiff. It was a short and relatively benign examination. Colohan appeared to contradict Wm Haire’s evidence on some minor points and admitted that he was aware that constant use of the “rays” might burn and that their use was “fraught” with danger. He said he had never said to Mrs McCullagh that he had never heard of an x-ray burn. He also said that he had attended the x-ray as a friend, and not to earn a fee.

## Redirect examination of Dr N Colohan

J Chambers then conducted a redirect examination of the defendant, Dr N Colohan. A redirect examination may be utilised by the party originally calling a witness, after a cross-examination has concluded. Generally, it is utilised as an opportunity to clarify or rebut any perceived issues raised in cross-examination. No new evidence may be introduced. The examination should be short and precise otherwise a long redirect will suggest the cross examination was the most informative for the triers of fact. It should perhaps, if possible, be dramatic as it is the last time the jury will hear directly from the defendant, unless of course he/she are defending themselves, and it will stick in their minds when it comes to deliberations. Colohan was asked by Chambers K.C. what he knew of Mr Haire’s expertise. Dr Colahan replied that as far as he was aware Mr. Haire was “the only X-ray operator in Connaught.”

An examination of a professional witness-to fact, a Surgeon McArdle from Dublin, then was conducted. McArdle had seen Thomas McCullagh (jnr) in April 1903 but had not examined him before referring him on to Mr Lewis to be “rayed”. He gave evidence that in his work he used a Davis X-ray machine and he had never seen a burn with this type. He stated that he normally gave an exposure of 30 min at 6 inches. In cross-examination by O’Shaughnessy K.C. McArdle then said he would have x-rayed the boy even if he had seen the burn.

## Examination of third defendant: Professors of Queen’s College Galway

The next witness called as a co-defendant was a Professor Kinkead one of the established Professors of the Queen’s College, Galway. Professor Kinkead had been the Professor of Midwifery and Gynaecology in Queen’s College Galway since 1876. There was a history of litigation involving Professor Kinkead and Dr Colahan so it was interesting that they were both appearing as co-defendants in the “Great Galway X Rays Case”. Colahan, had with the help of some “very recently”, paid-up “Life Governors” of the Galway County Infirmary managed to engineer his election as the Surgeon to the Infirmary. Professor Kinkead, who had pursued a long campaign to get the Infirmary to open up to the teaching of the medical students of Queen’s College sued the Infirmary alleging Colahan’s election as being an invalid election. Multiple legal actions followed. Colahan stayed in post until 1892 when a new Parliamentary Act changed the name and function of the infirmary to the Galway Hospital, allowing medical students to attend the hospital for the first time and delegating the appointment of permanent medical staff to the Local Appointment Board in Dublin. When these appointments were made they did not include Dr Colahan but did include Professor Kinkead and the other teaching Professors in the College. Colahan’s Professorship of Materia Medica In Queen’s College, Galway was not a full established professorship thus he was not granted automatic access to the Galway Hospital nor was he included as a co-defendant within the Queen’s College camp.

Questioned by Seymore Bushe K.C., acting for the College, Kinkead said he was a member of College Council and that he had examined the minute books for the duration of time since Mr Haire had come to work in the College. He could find no record in the minutes of Mr Haire being given formal permission to use the machinery. Kinkead also said he could find no record of any account from Mr McCullagh (snr) in relation to the matter being paid to the College. In cross-examination O’Shaughnessy K.C. for the plaintiff asked Professor Kinkead who has control of the equipment. Kinkead said each head of department was responsible for the equipment in their depart but ultimately answered to the Council of the Queen’s College.

Following Professor Kinkead there was an examination of Professor William Westropp Brereton, the Professor of Surgery in Queen’s College, Galway. W.W. Brereton had been a demonstrator in anatomy in Queen’s College Galway then became a district medical officer for 20 years in Oughterard, Co. Galway. He returned to the College take up the Professorship of Surgery in 1888. He was always to be found in his workshop imagining and fabricating new inventions. When asked by Gordon K.C., M.P. for the Queen’s College Brereton said he had sent many people to be x-rayed by Mr Haire and the pictures were always of good quality. He was not cross-examined.

At this point the only expert witness called for the Queen’s College was a Professor John A. McClelland. John McClelland was a graduate of Queen’s College, Galway winning the Gold Medal for Physics in 1892. After research in the Cavendish Laboratory in Cambridge he returned to Ireland to become Professor of Physics in the Jesuit controlled University College (later UCD) in 1900, based in Newman House on St. Stephen’s Green. In response to Seymore Bushe K.C. for the Queen’s College Professor McClelland gave evidence that he had examined the tube and coil used in the case and he had thought them in “good working order”. In cross-examination T O’Shaughnessy K.C. for the plaintiff asked Professor Mc Clelland about burns. He said he had never seen one that was the result of an X-ray application. O’Shaughnessy then enquired if he (McClelland) agreed with an earlier witness Dr O’Donnell and that x-ray burns were “largely mythical.” Professor McClelland replied, “in my experience I have never seen one.”

## Examination of Professor Anderson

The final co-defendant of the Queen’s College was Professor Alexander Anderson, wearing his two hats as Professor of Natural Philosophy (Physics), where the X Ray equipment was maintained and used, and as President of Queen’s College, Galway. Examined by Seymore Bushe K.C. for the College Professor Anderson explained that as he was the Professor of Natural Philosophy in the College he had the x-ray instruments in question under his charge. He also affirmed that he had given Haire permission to use the “X ray apparatus”. Somewhat disingenuously Anderson then said that he had not “authorised” Haire to charge a fee but that he had “advised it.” He subsequently had had nothing to do with the submitted account. He was then asked about his own practice in taking X-rays and he said he generally, used a distance of 5–6 inches and an exposure time of 20 −30 min.

Cross-examined by O’Shaughnessy K.C. for the plaintiff he asked Professor Anderson to clarify what he meant by advising Haire to charge a fee and whether it was part of a President’s Duty to do so. Anderson confirmed that he had “advised” Haire to send a fee and would do so again. O’Shaughnessy asked whether he as President would do so again, and Anderson replied that he would. He then opinioned to state that the experience gained by Haire in watching him and his previous assistant Dr Henry was, “sufficient to qualify him (Haire) as an x-ray manipulator.”

Mr Gordon K.C., M.P. then rose and asked the Judge, Gibson J to formally remove the College from the case. Gibson J said he would ask the special jury their opinion on the College’s liability, but he himself remained, “of the opinion there was no case against the College.”

The case for the co-defendants was declared completed and the Court rose.

On Wednesday February 10th, 1904, the 6th day of the “Great Galway X Rays Case”, before the closing statements by the barristers for the plaintiff and co-defendants were delivered there was a brief re-direct examination of Mrs McCullagh about her recollection of her interaction with Surgeon McCardle in Dublin. It transpired that from her communications with him after Mr Lewis had refused to conduct the “ray” that had McArdle seen the burn he would not have ordered an x -ray.

### Closing statements

The first of the closing statements that followed was made by Featherstonhaugh K.C. on behalf of the Queen’s College, Galway. [76] He summarised by saying that no “contract” between the plaintiff and “any single member of the governing body” had been entered into by the defendants. The “radiographs”, he concluded, “were merely taken as a friendly gesture to oblige the McCullaghs.”

Wylie K.C. on behalf of William Haire, said that his client had never “held himself out as a person open to employment for reward or otherwise”; that the McCullaghs were aware both of his “knowledge” of X-rays and his position in the College and that the fee paid was for the sole purpose of “defraying” expenses.

Chambers K.C. on behalf of Dr Colahan, argued that there was no case against that “gentleman”. If the McCullaghs had thought there was negligence by Dr Colahan they would have included him in their original suit, but his name was only added to the action after advice by counsel.

Mathewson K.C. on behalf of the plaintiffs, opened by stating that William Haire had been appointed to his position by the President of the College “pursuant” to the Charter of the College. His duties were therefore as an officer of the College under the control of the President. Mathewson K.C. then outlined the causation of x-ray burns been secondary to short distances and long exposures and that the “margin line of safety had not been observed in this case.” He concluded by declaring that the plaintiff had been “subjected to the rays by a person who had no proper skill in the matter.”

Following the closing arguments the case was adjourned for the day.

## 7th day of “Great Galway X Rays Case” trial: Judge Gibson’s charge to the special jury

The Court reconvened for the final time on the morning of Thursday February 11th, 1904. For the Galway contingent gathered near the No. 2 Court in the “Four Courts” there was a smattering of conversation while they waited. For some it was talk of the defeat in rugby of the Queen’s College by Old Galwegians 10 points to 7 the previous afternoon. For others it was sympathies for and shock at the untimely death of Dr MF Lydon, Medical Officer in Galway No 1 Dispensary District. For others it was extracting any gossip arising from the Drapers’ Ball held the previous Saturday in Galway’s Town Hall. Newspaper Editorials decrying the outbreak of the Russo-Japanese Conflict dominated the international news.

The Judge, Gibson J in his charge or instruction to the Special Jury reiterated his comments from earlier in the trial and instructed the special jury members that the College was not to be held in any way to blame and to remember that Mr Haire and Dr Colahan were not responsible for each other’s mistakes, if any were thought to have occurred.

In regard to the presence of expert witnesses at trial he noted that in an action for negligence that the import of their evidence must be considered carefully as “experts all differ in certain things” except that in this particular case they mostly had “agreed with each other” that the risk of x-ray burns was “infinitesimal” as against the possible effect of a foreign body in the knee. Gibson J also stated that if the jury found for the plaintiff (i.e. if negligence proven) then any award decided upon should be small. He then asked the jury to deliberate on a series of very specific and determining questions to address once they had reached their conclusions.

Finally, Gibson J explained to the special jury the elements of the action or tort of negligence as it was then understood. Although not reported in detail in the newspaper account he would have explained that the elements that they had be satisfied in an action for negligence with were the duty of care owed, breaches of that duty, and the necessary link between the breaches and the consequences. In instructing the special jury Gibson J would have leant heavily on the concepts of foreseeability and recklessness in negligence actions, that had been delivered in the relatively recent and very influential minority opinion of Lord Esher (then Brett M.R.) in *Heaven v. Pender* in 1883 in the English Court of Appeals. In the judgement Brett J had stated in his minority opinion, “Everyone ought to think... with regard to the safety of others who may be jeopardised by his conduct; and if, being in such circumstances, he does not think, and in consequence neglects, or if he neglects to use ordinary care or skill, and injury ensue, the law... will force him to give an indemnity for the injury.” This was an *obiter dicta* opinion that would underpin the denouement of the Tort of Negligence for years to come.

## The verdict

The Special Jury retired to deliberate about 1.30 pm on the afternoon of February 11th,1904 and in very quick time, around 3 pm, returned to courtroom. Having confirmed they had reached a verdict Justice Gibson posed aloud the questions he had previously presented them with before they retired for their deliberations:

- **Justice Gibson (*JG***): Q1. Were the Queen’s College Galway, and Haire, or one and which of them, employed for reward to photograph by X-rays in December 1902, and in April 1903?
- **Jury Foreman (*JF*):** *Haire was so employed. College was not.*
- ***JG***: **Q2.** Was the sore caused by the x-rays? ***JF***: *Yes.*
- ***JG***: **Q3**. Were the rays negligently applied as regards:
  - Distance? ***JF***: *No.*
  - Duration of each exposure? ***JF***: *No.*
  - Consecutive multiplication of exposures? ***JF***: *No.*
  - The Type of Machine? ***JF***: *No.*
- ***JG***: **Q4.** Was the sore caused by the operation –
  - Of December 15th? ***JF***: *Cannot say*.
  - Of December 26th and 27th? ***JF***: *Cannot say which*.
  - Aggravated by these in April? ***JF:*** *No*.
- ***JG: Q5.*** (a) Did the operation of Dr Colahan, for which Haire is not responsible, contribute to the sore? ***JF***: Yes
  (b) Did the operation of Haire, for which Dr Colahan is not responsible, contribute? ***JF***: Yes
- ***JG***: **Q6.** Was Haire **negligent** in applying the rays**? JF**: **No.**
- ***JG: Q7. ***Before the rays were applied in April was–
  - Dr Colohan **negligent** in not diagnosing the sore as caused by X-rays? **JF**: **No**.
  - Was Haire as **negligent**? **JF**: **No**.
- ***JG: Q8***. (a) Was Dr Colohan **negligent** in applying the rays in April after the sore appeared? ***JF***: **No**.
  (b) Was Haire as negligent? ***JF***: **No**.
- ***JG:*** **Q9**. To what damages, if any, is the plaintiff entitled? ***JF***: **None**.

**Verdict:** On these findings ***a verdict was entered for the defendants*** and thus ended the earliest Irish civil trial for medical negligence where X-rays were employed.

## Conclusion

*For the strangers came and tried to teach us their way*

*They scorn’d us just for being what we are;*

*But they might as well go chasing after moonbeams*

*Or light a penny candle from a star*

The above lines come from the 4th verse of the song “*Galway Bay*”. The lyrics were penned in 1947 by Dr Arthur NW Colahan, the son of Professor Nicholas W Colahan the co-defendant in the “Great Galway X Rays Case” trial. There is no doubt that Dr Colohan demonstrated a heightened duty of care to Thomas McCullagh (jnr) and that there was at trial no demonstrable breach of that duty. [77] Hence the “no” verdict of negligence was appropriate. Equally William Haire had performed all his professional duties in conducting and arranging for x-rays to be taken, according to the scientific information available to him in 1902 in regard to application of x-rays, and the issues over shady payments did not undermine the special jury’s recognition of that duty being satisfied.

In contrast to the sentiment of the song the “strangers” in the “Great Galway X Rays Case” action were the expert professional witnesses opinioning evidence concerning the new “moonbeams” known as x-rays. Their expertise, early as it was in the development and application of the technology, was significant and persuasive in the trial. The *Galway Express* in a banner headline tried to give a sense of a significant conflict of expert witness evidence but in essence, as reported, there was no conflict but instead a reasonably accurate narrative and interpretation of evolving object-subject distances, exposure times and quality of vacuum tubes in various locations. There was less agreement about the risks and incidence of x-ray burns and this disagreement would make finding for the plaintiff difficult. There was however, where burns were concerned, certain disingenuous economies of truth where two of the expert witnesses, Lane Joynt and Hall-Edwards, did not specifically disclose at trial that they themselves had been burnt by x-rays and left with significant sequelae.

The impression reading the accounts of the “Great Galway X Rays Case” trial is that the boy-plaintiff’s Thomas McCullagh’s burns were somehow dismissed as imaginary. John Townsend Pitkin (1858–1935) is considered an “American Martyr to Radiology”. In a communication to American Roentgen Ray Society in 1903 regarding x-ray burns, Pitkin wrote: “For a description of the pain and suffering, no language, sacred or profane, is adequate. The sting of the honey-bees or the passage of a renal calculus is painful enough, but they are comparative pleasures, because being paroxysmal they have a time limitation. Extreme tenderness to the slightest touch. Hot and cold waves and flashes, warmth, tingling, pricking, throbbing, stinging, crawling, boring and burning sensations, as if the parts were on fire…”. [78]

In essence the outcome of the “Great Galway X Rays Case” was very similar to the first US X-ray burn negligence case from a year earlier in 1903, another action not proven. In that case there was a radiological search for a lost gold tooth crown and a total of 5 × 30 min exposures were made with no evidence of the crown on the photographs. Two weeks later the patient in the US case, a man called Henslin developed a deep ulcer on his back that became recurrent. Henslin sued his medical carers who administered the x-rays but in the trial in March 1903 the evidence of the expert witness called on his behalf was excluded because he was a physicist and not a medical doctor. Minnesota only allowed one expert witness to appear at a negligence trial. Henslin lost the case. [79] On appeal the Minnesota Supreme Court determined that the expert evidence should have been admitted and a retrial was ordered. This retrial in March 1904, a month after the Galway verdict, was also lost by the plaintiff.

Even if there was negligence, as we currently understand it, in the delivery of x-rays in Queens College, Galway in late 1902, the clinical application of Wilheim Konrad Röntgen’s 1895 discovery of X-rays was still at a very nascent part of its development. The characteristics of the cathode–anode vacuum tubes and globes, the voltage required to effect the optimum electron discharge and the ideal photographic plate technology were all rapidly evolving. The setting of exposure times and subject-object distances, with the new technique and the determination of application protocols and recording outcomes – negative and positive – were very much a work in progress as was evident from the expert evidence examined. It was another 10–15 years before negligence suits became far more common in Radiology but more often by then they were for misdiagnoses than for any foreseeable direct injury caused [80] .

These facts, from the Special Jury’s perspective, meant that where they were concerned there was no fixed post on which to hoist the petard of a negligent act, a fact emphasised to the jury in his charge by the trial judge, Gibson J. X-Ray expertise in 1904 would have been considered as someone who was reasonably *au fait* with the practical workings of an X-ray machine or who could demonstrate he was up-to-date with all the scientific literature pertaining to X-rays and perhaps x-ray burns.

Defending negligence cases in Radiology still creates a significant issue for defence bodies such as the Medical Protection Society and there are still significant professional concerns over occupational exposure to radiation with fluoroscopists still being exposed to higher doses [81].

The use of expert scientific evidence in both criminal and civil trials has continued to evolve over time. In the main the duties of an expert witness have been established in case law and then subsequently regulated in statutory law. There are many professional and personal dangers associated with the nature and impact of opinion evidence presented and in some other jurisdictions experts themselves are being sued for negligence if their evidentiary duty fails to meet the require standards. Peter Charleton, a judge of the Irish Supreme Court and an adjunct Professor of Criminal Law and Criminology in the University of Galway and Ivan Rakhmanin, a judicial assistant in the Supreme Court, wrote in an article in 2023,

“*Experts are indispensable to the administration of justice. Why? Because litigation ranges way beyond what judges or juries comfortably deal with as the facts of everyday life. Whether it is the diseases of the mind, or the chemical reactivity of pharmaceuticals or the obviousness of a contended-for inventive step in a patent case, without the assistance of experts, courts would be vastly under-equipped in making decisions of fact. But, here there is a real danger: that of over-reliance, or even of the surrender of the authority of the judge to experts; to those paid by litigants to testify helpfully on their behalf. Recognising that danger, the analysis of the law of evidence and the practical approach of the courts to expert testimony should both confine the use of experts within definable boundaries and also require judges to equip themselves with that ordinary distance from witnesses that will enable judicial independence to be seen to be upheld.” *[82]

In recent years in Ireland there has been a determined effort to reign in and regulate on a statutory basis the use and duty of Expert Witnesses. [82–84]

It was immediately recognised that in losing the 7-day “Great Galway X Rays Case” the costs of the trial for the McCullagh family was enormous. The cost of all expert witnesses alone was estimated at 100 guineas each as a retainer (€18,804 in today’s value) and 20 guineas per day each thereafter for the days spent attending the trial. In addition, there were 14 counsel representing plaintiff and defence as well as 4 local solicitors and of course the single guinea paid to each member of the Special Jury. King’s Counsels as a minimum got 50 guineas as a retainer and 25 guineas *per diem* thereafter in addition to other consultation fees. A King’s Counsel could negotiate any fee they felt appropriate or get away with. It was estimated by the *Galway Express* reporter that the total costs of the action could reach a minimum of £2500 that would then have to be paid by the losing side, in this case the McCullagh plaintiffs. This amount would come in today’s value, adjusting for inflation, to approximately €447,638. As was noted above Wm Haire’s salary in 1904 was about £58 pa whereas a full-time established professor in the Queen’s Colleges of the Royal University of Ireland earned about £700 pa. (equivalent to about €125,339 in today’s value) [85].

The family seem however to have surmounted the financial implosion losing the trial would have created for them. In the 1911 census, 7 years after the trial, the McCullagh family remained in Galway and Thomas H. McCullagh the plaintiff, then aged 14 and described as a scholar, was living with his 5 siblings, his mother Jane F., aged 42 and his father Thomas A., aged 50 at Williamsgate Street in Galway. They appear to still have their stationary shop and a roof over their heads and indeed the shop had expanded to start selling phonographic records as well Of note. Thomas (jnr)’s sister Ida, who gave evidence in the Galway case, was said to be 14 in 1904 at the time of trial yet in 1911 her age was given as 19 years. She had lost two years along the way, perhaps another side effect of those “rays”!

In the end, at least to the end of the trial, despite multiple x-rays there was only one report that suggested the actual presence of a needle fragment in Thomas H. McCullagh’s right knee, and this was not confirmed in subsequent X-ray exposures. There also was no notation of it ever having been found or ever having spontaneously discharged from the wound before the trial started. This perhaps was the most important factor in failing to find negligence in the management of the case; a failure of *habeus corpus* of a needle fragment you might say.

In the “Great Galway X Rays Case” trial an x-ray photograph pertaining to the quality of X rays taken by the co-defendant Wm Haire was entered into evidence despite the objections of O’Shaughnessy K.C. for the plaintiff over who had developed the negative. The judge determined admissibility on authorship of the image. Of interest, the first use of an x-ray photograph, as a physical scientific image, as probative evidence in a medical negligence case in the USA was in *Smith v. Grant* in Colorado in 1896. The admission of the x-ray images into evidence in a negligence trial was seen as transformative for US law and laid the ground for both the seminal 1923 *Freye* case and affirming 1995 *Daubert* ruling for the validation and admission of scientific and medical evidence. [86]

The Natural Philosophy or Physics Department in Queen’s College, Galway stopped performing X-rays on notice of the legal suit by the McCullagh’s in the Autumn of 1903. Following the trial the X-ray equipment, which had been on display and used in evidence in the trial, was transferred to the Galway Hospital on Prospect Hill where Mr William Haire continued to operate the equipment on a fee per item basis. How long he did this for is uncertain. His name does not appear in 1911 census anywhere in Galway and it is easy to speculate whether he died in the meantime from x-ray related radiation damage from his work or simply returned home to Mayo. In 1922 and 1924 respectively the Galway Hospital and Galway Surgical Hospital (the former County Infirmary) transferred to and replaced the Central Hospital located within the old 1841 Workhouse. A new department was formed and new X-ray equipment purchased for £17 (€1522 today’s value). Dr Conor O’Malley was the ENT surgeon and Radiologist and his formal appointment in 1928 as a Consultant Radiologist by the Local Appointments Board was the first such appointment to a local authority hospital in Ireland [87]. 

The days of the hobbyist radiologist in Galway were over.
